# Targeted suppression of glutaryl-CoA dehydrogenase by lentivirus-mediated SHRNA and excessive intake of lysine induce the apoptosis of rat striatal neurons

**DOI:** 10.1186/1687-9856-2013-S1-P172

**Published:** 2013-10-03

**Authors:** Gao Jinzhi, Fu Xi, Zhang Cai, Gao Hongjie, Luo Xiaoping

**Affiliations:** 1 Tongji Hospital, Tongji Medical College of Huazhong University of Science and Technology, China

**Keywords:** glutaric aciduria type 1, glutaryl-CoA dehydrogenase, lysine, short hairpin RNA, apoptosis

## Aims

In Glutaric aciduria type 1 (GA1), Glutaryl-CoA dehydrogenase (GCDH) deficiency has been responsible for accumulation of glutaric acid (GA) and striatal degeneration. However, the mechanisms by which GA1 induces striatal degeneration remain unclear. In this study, we aimed to establish a novel neuron model of GA1 and explore the underling mechanisms of striatal lesion.

## Methods

Four short hairpin RNA (shRNA) sequences targeting the GCDH gene (NM_001108896) were designed to construct four recombinant lentiviral vectors. The effectiveness of gene silencing in rat striatal neurons was detected by real-time reverse transcription polymerase chain reaction (RT-PCR) and Western blotting techniques. GCDH deficiency neurons (GCDH^-/-^ neurons), neurons transfected with negative control virus (NC neurons) and not intervention neurons (C neurons) were all incubated with lysine for 24h in concentrations of 0mmol/L, 5mmol/L, 10mmol/L respectively. The viability was measured with 3-(4, 5-dimethylthiazol-2-yl)-2, 5-diphenyl-tetrazolium bromide (MTT). The apoptosis of the neurons were detected by Hoechst33342 and PI. Tetramethylrhodamine methyl ester (TMRM) was used to determine the change of mitochondrial membrane potential. The expression of caspase-3, 8, 9, Bax and Bcl-2 were examined by RT-PCR and Western blotting.

## Results

The efficiency of gene silencing of lentivirus-mediated shRNA4 was up to 60%, compared with the parental and control groups. The viability of C neurons, together with mitochondrial membrane potential and expression of caspase-3, 8, 9, Bax/Bcl-2 was not influenced by lysine, even when the concentration was 10mmol/L. The viability of NC neurons was significantly higher than GCDH^-/-^ neurons, when with 5mmol/L lysine interference. When without lysine, there is no difference between the two. Moreover, 5mmol/L lysine could induce GCDH^-/-^ neurons apoptosis, and 10mmmol/L lysine could induce NC neurons apoptosis. In these apoptotic neurons, the mitochondrial membrane potential decrased, the expressions of caspase-3, 8, 9, Bax all increased significantly and Bcl-2 expression decreased, compared to normal cells.

## Conclutions

These results indicated that targeted suppression of GCDH by lentivirus-mediated shRNA and excessive intake of lysine may be useful as a neuron model for the study of GA1. It also showed mitochondrial apoptotic pathway may be involved in the GA-induced striatal lesion.

